# Sphingolipids and Chronic Kidney Disease

**DOI:** 10.3390/jcm13175050

**Published:** 2024-08-26

**Authors:** Zrinka Šakić, Armin Atić, Slavica Potočki, Nikolina Bašić-Jukić

**Affiliations:** 1 Vuk Vrhovac University Clinic, Dugi dol 4a, 10000 Zagreb, Croatia; 2 Division of Nephrology, Arterial Hypertension, Dialysis and Transplantation, University Hospital Center Zagreb, 10000 Zagreb, Croatia; 3 School of Medicine, University of Zagreb, 10000 Zagreb, Croatia

**Keywords:** sphingolipids, chronic kidney disease, ceramides, sphinogosine-1-phosphate

## Abstract

Sphingolipids (SLs) are bioactive signaling molecules essential for various cellular processes, including cell survival, proliferation, migration, and apoptosis. Key SLs such as ceramides, sphingosine, and their phosphorylated forms play critical roles in cellular integrity. Dysregulation of SL levels is implicated in numerous diseases, notably chronic kidney disease (CKD). This review focuses on the role of SLs in CKD, highlighting their potential as biomarkers for early detection and prognosis. SLs maintain renal function by modulating the glomerular filtration barrier, primarily through the activity of podocytes. An imbalance in SLs can lead to podocyte damage, contributing to CKD progression. SL metabolism involves complex enzyme-catalyzed pathways, with ceramide serving as a central molecule in de novo and salvage pathways. Ceramides induce apoptosis and are implicated in oxidative stress and inflammation, while sphingosine-1-phosphate (S1P) promotes cell survival and vascular health. Studies have shown that SL metabolism disorders are linked to CKD progression, diabetic kidney disease, and glomerular diseases. Targeting SL pathways could offer novel therapeutic approaches for CKD. This review synthesizes recent research on SL signaling regulation in kidney diseases, emphasizing the importance of maintaining SL balance for renal health and the potential therapeutic benefits of modulating SL pathways.

## 1. Introduction

Sphingolipids (SLs), as bioactive signaling molecules, are integral to various cellular processes such as cell survival, proliferation, migration, and apoptosis [1]. The sphingoid base with a bonded fatty acid forms the structural basis of sphingolipids. Based on the type of attached substituent, sphingolipids can be classified into several categories, including ceramides, glycosphingolipids, and sphingomyelins. Among all SLs, ceramides, ceramide-1-phosphate (C1P), sphingosine, and sphingosine-1-phosphate (S1P) are the most well-known bioactive SLs. An imbalance in sphingolipid levels can be detrimental to cellular integrity. Abnormalities in sphingolipid concentrations are associated with numerous diseases, including renal disorders. Chronic kidney disease (CKD) is a growing global health challenge, affecting approximately 10% of the population worldwide [2]. This condition is characterized by a gradual decline in kidney function over months or years, often leading to end-stage renal disease (ESRD), where dialysis or kidney transplantation becomes necessary for survival. CKD is associated with a high risk of cardiovascular disease, which is a leading cause of death in these patients. The prevalence of CKD is strongly linked to the increasing incidence of diabetes and hypertension, which are the two most common causes of the disease. As the global population ages and the rates of diabetes and hypertension continue to rise, the burden of CKD is expected to grow substantially. CKD not only impacts the health and quality of life of individuals but also places a significant strain on healthcare systems due to the high costs of treatment and the management of associated complications. Recent research into the molecular mechanisms underlying CKD has demonstrated the pivotal role of SLs in maintaining renal function. These molecules can alter the glomerular filtration barrier by modulating the activity of podocytes, which are critical components of this barrier [3]. Alterations in sphingolipid metabolism have been implicated in the pathogenesis of CKD, influencing cellular stress responses, inflammation, and fibrosis [1]. Understanding these lipid pathways offers new insights into the mechanisms driving CKD progression and opens potential avenues for novel therapeutic strategies aimed at modulating sphingolipid metabolism to improve kidney health and prevent disease advancement. Importantly, a large body of knowledge regarding SLs stems from studies on animal models, chiefly on rodents, which do have some differnces regarding ther sphingolipidomic profile when compared to humans. Specifically, they have lower SL levels; however, for some species, such as ceramides or a subtype of sphingomyelin, no differences to human plasma were found, and rodents, specifically hamsters and mice, are considered relevant models to reflect the human setting [4]. This review synthesizes recent research on regulating sphingolipid signaling in kidney diseases and explores the potential of SLs as biomarkers for early detection and prognosis of chronic kidney disease (CKD).

## 2. Bioactive Sphingolipid Classes and Their Metabolism

The metabolism of sphingolipids is complex due to numerous enzyme-catalyzed processes that result in distinct signaling molecules in various body regions and within a single cell. 

**Figure 1:**
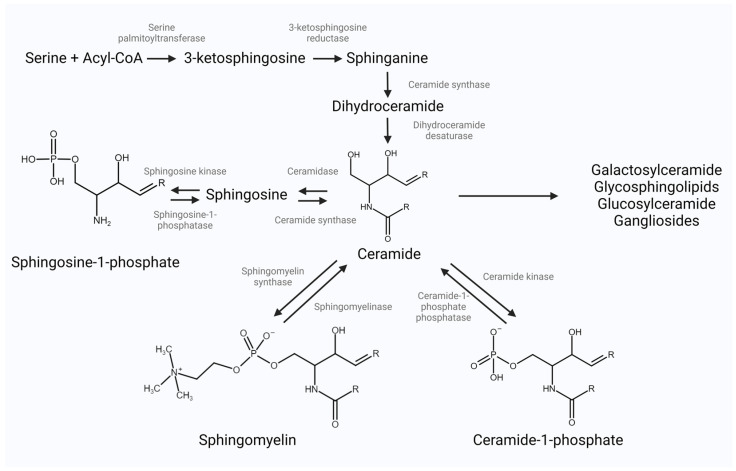
Sphingolipid synthesis pathways, created with Biorender.com. Note: various enzymes involved in the formation of complex sphingolipids are not presented here.

The human body may synthesize SLs through de novo and salvage pathways, with the “ground” molecule being the sphingoid base, which produces other simple sphingolipids and their intermediates [5]. Ceramide is the common de novo and salvage pathways molecule and the central molecule for complex sphingolipid synthesis. Newly synthesized ceramide can then undergo conversion by different enzymes to any of the possible pathways to form one of the main bioactive terminal products, whose main representatives are sphingosine-1-phosphate, sphingomyelin, and ceramide-1-phosphate (Figure 1) [6]. Each of the subclasses is involved in different cellular pathways and diverse roles across the organism.

### 2.1. Ceramide

The two pathways mentioned earlier can form ceramide as the building block of other sphingolipids. In the de novo pathway, its formation is triggered either by a metabolic overload of serine or by a stress stimulus, such as heat, tumor necrosis factor, cannabinoids, oxidation, glucocorticoids, or chemotherapeutics [7,8]. It begins in the endoplasmic reticulum with the formation of a sphingoid backbone by condensation of serine with palmitoyl-CoA catalyzed by a serine palmitoyl transferase [9]. Further along the pathways, a family of ceramide synthases adds a variable acyl group to form the dihydroceramides. Six described ceramide synthases (CERS1-6) have specific substrate specificity and tissue distribution [6]. The variance of CERS enzymes and their products allows for a diverse regulatory role of sphingolipids in different states. For example, CERS2 is ubiquitously expressed, particularly in the kidney and liver, and the products of this enzyme (very long-chain ceramides) are considered protective [10].

On the other hand, CERS5 and 6 are upregulated in stress states and promote the formation of long-chain C16 acyl groups implicated in metabolic dysfunction [8,11]. However, it is still uncertain whether the properties associated with ceramide are sensitive to the different lengths of acyl chains. However, several CERS types may allow advantages in specific cellular functions in various tissues [6]. In the salvage pathway, ceramide may be formed through the breakdown of sphingomyelin through the action of sphingomyelinase, or by the degradation of complex sphingolipids through a series of sequential hydrolysis. In the kidney, ceramides are twice as abundant in the cortex than in the medulla, while transcriptomic studies show a surprising heterogeneity of ceramide-related gene expression across kidney cell types. For example, CERS6 shows high expression in the podocyte, while *SPTLC2*, a gene encoding serine palmitoyltransferase, has high expression in the loop of Henle. In contrast to prior findings, CERS2 expression was found to be low in isolated cell clusters using single-nucleus RNA sequencing [12,13]. Further research is required to elucidate the spatial distribution of ceramides as well as the effects of differential expression of ceramide-related genes and their products across cell types in kidneys.

### 2.2. Sphingosine-1-Phosphate

When ceramide is hydrolyzed by a ceramidase to sphingosine, it can then be phosphorylated by one of the sphingosine kinases to form sphingosine-1-phosphate (S1P). There are two known isoforms of sphingosine kinases, 1 and 2, and they display differential expression in different compartments and they have different functions. Sphingosine kinase 1 (SphK1) is expressed in the cytosol and it may exert anti-apoptotic functions in renal mesangial cells by increasing S1P levels [7,14]. On the other hand, sphingosine kinase 2 promotes apoptosis.

S1P exerts its functions via one of the five known S1P receptors—S1PR1-5. Differential signaling of S1P is determined by the type of S1P receptor and its localization [15]. In mammalian cells, S1P can be irreversibly degraded by S1P lyase into phosphoethanolamine and hexadecenal, or it can be converted back to sphingosine by phosphatases [16]. 

### 2.3. Ceramide-1-Phosphate

Ceramide can also be directly phosphorylated to produce ceramide-1-phosphate (C1P) by the ceramide kinase (CERK), but also through mechanisms independent of CERK, as has been shown in studies in CERK knockout mice [17,18]. C1P is a substrate for non-specific lipid phosphatases, which play a role in extracellular S1P signaling [6,19].

### 2.4. Complex Sphingolipids

Complex sphingolipids are a diverse group of sphingolipids with many structural, signaling, and regulatory roles across different tissues and cell types. There are three major groups of complex sphingolipids formed by the action of three chief enzymes-ceramide galactosyltransferase, glucosylceramide synthase, and sphingomyelin synthase. Galactosylceramides are formed through the action of ceramide galactosyltransferase, an enzyme present in neural tissue, intestines, and the kidneys. Their function is highly important for the function of oligodendrocytes and overall myelination in nerve tissues. These sphingolipids can also serve as precursors for the formation of other molecules, such as sulfatides. Glucosylceramide and its derivatives are synthesized by the glucosylceramide synthase and represent an essential sphingolipid in mammalian development, as it is a precursor for the majority of all glycosphingolipids [20]. Sphingomyelin is the most abundant complex sphingolipid in humans, and it is synthesized from ceramide by sphingomyelin synthase. 

## 3. Sphingolipid Role in Normal Renal Function

Sphingolipids in normal kidneys serve the function of maintaining the structural integrity of cellular membranes; however, they also serve roles as signalling molecules in various physiological functions. The glomerular filtration barrier (GFB) consists of podocytes, the glomerular basement membrane (GBM), and glomerular endothelial cells. Podocytes are specialized epithelial cells with foot processes that interlock to form filtration slits, bridged by the slit diaphragm. The integrity of podocytes, particularly their actin cytoskeleton, is essential for GFB function [21]. The sphingolipid balance plays a significant role in kidney diseases. Although it is not fully understood how disruptions in sphingolipid metabolism cause podocyte damage, various disorders emphasize the crucial role of sphingolipids in maintaining podocyte health. Sphingolipids are an integral part of the regulation of disorders associated with oxidative stress, and they regulate the production of reactive oxygen species (ROS) [22,23]. Their role is regulation of apoptosis, stress response, and cell proliferation, and individual sphingolipids may have opposing roles, for example, ceramide is considered a pro-apoptotic factor while sphingosine-1-phosphate is anti-apoptotic. The exact role of various sphingolipids in maintaining normal kidney function is not fully elucidated, and our knowledge mainly stems from research regarding their role in inflammation and their disorders in various disease processes, such as chronic kidney disease, diabetic kidney disease, and glomerular diseases. Studies concerning the physiologic role of sphingolipids in kidney function are mainly focused on their effects on blood pressure homeostasis via effects on local circulation and natriuresis.

The chief role of ceramide is the induction of apoptosis, and under normal conditions, the intracellular concentration of ceramide is kept low until a harmful event occurs, such as the administration of a nephrotoxic drug, irradiation, exposure to bacterial toxins, or hypoxia [24,25,26,27]. Under such conditions, ceramide metabolism is impaired and leads to the accumulation of ceramide, which then induces apoptotic processes chiefly via altering mitochondrial outer membrane permeability, resulting in ROS generation, release of cytochrome C, activation of caspase, and ultimately, cell death. Interestingly, the two isoforms of S1PK have opposing roles; SphK1 promotes cell survival whereas SphK2 promotes apoptosis in kidney injury. The SphK1/S1P axis promotes cell survival and acts protectively in various kidney injury types, such as the administration of cisplatin, hypoxic injury, and other pathophysiologic mechanisms integral to the development of chronic kidney disease [28,29,30]. The severity of oxidative stress determines the cellular response, and severe oxidative stress inhibits SphK1 activity, thereby governing the response away from cell proliferation [31]. Taken together, the current opinion prevails that the ceramide-S1P homeostasis serves as a rheostat determining cell fate following oxidative injury.

Other than their key role in cell response to injury, sphingolipids are shown to have a physiological role in the regulation of circulation and natriuresis. Endothelial cells, in response to various rheological and chemical stimuli, release vasoactive factors, one of which is S1P [32]. S1P, acting through S1PR1, is a potent activator of the endothelial nitrous oxide synthase (eNOS). It is of note, however, that activation of S1PR2/3 induces vasoconstriction in vascular smooth muscle cells of rodent arteries, which may be counteracted by the S1PR1/3 mechanism by eNOS activation [33]. S1PR1 is 16 times more abundant than S1PR3, and in S1PR3-negative mice the vasorelaxation effect of S1P was absent, indicating the contribution of S1PR3 to the effect of S1PR1 stimulation [34]. Further studies are required to fully elucidate the mechanisms by which these receptors regulate vascular tone; however, it appears that endogenously created S1P acts mainly as a vasorelaxation agent in arterial resistance vessels. When administered exogenously, however, S1P constricts intrarenal arteries and reduces renal blood flow in mouse models [35]. This reduction in renal blood flow is associated with a transient increase in diuresis, natriuresis, calciuresis, and kaliuresis [36]. The decrease in medullary blood flow and increase in natriuresis is an effect also observed with the intravenous administration of fingolimod—an S1PR1 agonist [37]. The use of S1P agonists creates transient hypotension, particularly in animal models with acute or chronic hypertension [32,38]. The exact role of the subtypes of S1PRs in the pathogenesis of diseases such as hypertension remains unclear; however, findings indicate a possible therapeutic site for cardiovascular diseases. Deepening the role of sphingolipids in hypertension are findings of increased ceramide concentration in blood vessels as well as plasma of hypertensive mouse models [39]. Sphingolipids have also been shown to regulate the function of ion transporters in the kidney, such as the basolateral Na/K ATP-ase by ceramide-1-phosphate and the Ca^2+^ ATP-ase by ceramide in proximal tubular cells [40]. Further importance to this regulation was brought by findings of elevated ceramide concentrations after intracellular calcium dysregulation during renal injury [40,41]. Results of all these studies show that sphingolipid metabolism may be a target for the treatment of glomerular pathologies and complex metabolic disorders such as arterial hypertension.

Glycosphingolipids and gangliosides are abundantly expressed in renal cells and serve many structural, functional, and signaling roles. In particular, glycosphingolipid interactions regulate the partitioning of structural proteins and receptors, such as G-protein-coupled receptors and receptor tyrosine kinases, including insulin and epidermal growth factor receptors [42,43]. Glycosphingolipids are particularly abundant in epithelial cells of renal tubules [44]. Disorders of their expression are associated with a wide spectrum of renal disorders, such as storage diseases (e.g., Fabry disease), polycystic kidney disease, renal cancer, diabetic nephropathy, and glomerulonephritides [45]. Within podocytes, sphingolipid composition is integral for the function and maintenance of the glomerular filtration barrier. Different sphingolipid species have been identified as key components for the normal filtration process, and their dysregulation is implicated in various disorders associated with podocyte damage (Table 1), such as ceramide accumulation in Farber disease, S1P accumulation in S1P lyase deficiency, and sphingomyelin accumulation in Niemann-Pick disease, or accumulation of complex sphingolipids such as glucosylceramide or globotriaosylceramide in Gaucher and Fabry disease, respectively [46,47].

## 4. Role of Sphingolipids in the Pathogenesis of Chronic Kidney Disease

Chronic kidney disease (CKD) is identified as kidney damage or a decreased glomerular filtration rate (GFR) for over three months, and it is categorized into stages according to GFR levels [61]. CKD from all causes results in structural and histologic changes in all parts of the kidney, glomeruli show fibrosis, atrophy of the tubules ensues and there is interstitial infiltration by inflammatory cells as well as sclerosis of the renal vasculature. Whatever the mechanism, injury to one part of the kidney induces disease and injury of other areas as well [62]. Among these injurious processess, CKD is associated with an inflammatory state marked with an increase in proinflammatory cytokines, such as transformin growth factor ß (TGF-ß), interleukin-6, and others, which drive inflammation and are associated with higher levels of oxidative stress [63]. CKD is a common term for a decrease in renal function from all causes, and encompasses all stages from a mild reduction in function to complete renal failure. Sphingolipid disorders are implicated in a large number of pathologies leading to CKD, in some as drivers of the pathology, such as in some accumulation diseases, while in others they are a part of larger inflammatory, structural, and functional derangements. Selected sphingolipid disorders in various CKD etiologies are presented in Table 1. Sphingolipids, as mentioned earlier, play a critical role in the regulation of ROS production and levels of S1P and ceramides determine cellular fate. The renin–angiotensin–aldosterone system (RAAS) plays an important role in the development of CKD through different mechanisms, ranging from increased plasma protein filtration due to increased intraglomerular pressure but also through increased production of ROS, induction of TGF-β, and worsening renal injury and fibrosis through direct effects of angiotensin II and aldosterone [64]. All these effects put the RAAS in the central position in the development and progression of CKD, and RAAS blockade has been one of the mainstays of CKD treatment. SLs and the RAAS are interconnected at various levels, and SLs play important roles as second messengers for different functions of the RAAS. For example, in studies with sphingosine kinase knockout mice angiotensin II-induced hypertension was lower in SphK1 knockout mice and significantly lower or even absent in SphK2 knockout mice [49]. Furthermore, S1P is associated with an increased baseline plasma renin activity and through its effects on the Rho–kinase pathway is connected to the effects of angiotensin II, but also some medications, such as hydrochlorothiazide [65]. Ceramide, C1P, sphingosine, and S1P are also second messengers in the process of synthesis and release of aldosterone, although not all the mechanisms are yet fully elucidated [66]. Ceramide is also one of the second messengers involved in the angiotensin II AT2 receptor signalling, with the stimulation leading to apoptosis, and may also be involved in the AT2 receptor-mediated vasodilation [48]. Other risk factors for CKD, such as hypertension, diabetes, coronary disease, and others are linked to disturbances in the sphingolipid profile. Although dyslipidemia is linked to the onset of CKD, there has been limited research on specific circulating lipid molecules and their association with CKD risk. A recent study found that higher levels of various lipid classes, including sphingolipids, were linked to an increased risk of CKD, regardless of age, sex, body mass index (BMI), diabetes, or hypertension [67]. Sphingolipids in the blood are carried by different lipoproteins: low-, intermediate-, very low-, and high-density Apo B-containing lipoproteins (LDL, IDL, VLDL, HDL) along with other lipids like phospholipids, triglycerides, and cholesterol [68]. Common sphingolipids in the blood include sphingomyelin, glycosphingolipids, and ceramide. The bioactive molecule S1P is mainly bound to HDL particles, especially HDL3, and about a third of S1P is carried by serum albumin [69]. Bound S1P represents the bioactive form of S1P and mediates the protective actions of HDL, such as positive effects on the endothelium and vasorelaxation [34,70,71]. Studies indicate that its positive properties on the endothelium, by mitigating the inflammatory response to tumor necrosis factor-alpha (TNF-α), are only exhibited in the HDL/ApoM-bound form. Indicative measurements have shown that patients with chronic kidney disease have lower concentrations of S1P in HDL compared to healthy subjects [72]. 

In CKD, the role of ceramides is emphasized in promoting renal fibrosis and inflammation. Ceramides activate pro-fibrotic pathways, including TGF-β signaling, which leads to extracellular matrix accumulation and fibrosis. Additionally, ceramides impair mitochondrial function, further contributing to renal cell injury and the progression of CKD. Podocyte injury contributes to the progression of diseases like diabetic kidney disease (DKD), focal segmental glomerulosclerosis (FSGS), Alport syndrome, IgA nephropathy, and lupus nephritis (LN) [21]. Mallela et al. described how hyperglycemia leads to increased synthesis of ceramides in DKD. Elevated ceramide levels in the kidney contribute to podocyte apoptosis and dysfunction, which are critical in the development of proteinuria and glomerulosclerosis. Their study also highlights the role of glycosphingolipids, such as gangliosides, in modulating insulin signaling pathways, which exacerbates renal damage in diabetes [21]. Moreover, the dysregulation of SLs in acute kidney injury (AKI) is primarily driven by the accumulation of ceramides. In AKI, factors such as ischemia-reperfusion injury or nephrotoxicity lead to increased ceramide production through the activation of enzymes like sphingomyelinases. This ceramide accumulation induces mitochondrial dysfunction, promotes inflammation, and triggers apoptosis in renal cells, contributing to kidney injury [13]. Conversely, S1P is essential for normal podocyte function [55]. In support of this, radiation treatment has been shown to reduce S1P levels and induce podocyte injury, whereas administering S1P to irradiated podocytes protects them from radiation-induced damage by preventing cytoskeleton remodeling [73]. Likewise, lupus nephritis is associated with altered sphingolipid metabolism, particularly in immune cell regulation. Dysregulated S1P signaling affects immune cell trafficking and inflammation, leading to immune complex deposition and glomerular injury [21].

Focal segmental glomerulosclerosis (FSGS) is the main cause of nephrotic syndrome in adults [74]. The study conducted by Yoo et al. investigated the role of the enzyme sphingomyelinase-like phosphodiesterase 3b (SMPDL3b) in podocyte injury in glomerular diseases, including FSGS. The researchers found that the expression levels of SMPDL3b in podocytes influence how these cells respond to injury. Specifically, higher levels of SMPDL3b were associated with resistance to injury, while lower levels were linked to increased susceptibility to damage. The findings suggest that SMPDL3b could be a critical factor in determining the severity of podocyte damage in diseases like FSGS, potentially offering a target for therapeutic intervention [75]. Likewise, the study by Fornoni et al. found that rituximab, a monoclonal antibody typically used to treat B-cell malignancies, helps to stabilize podocyte function and prevent injury, suggesting that its therapeutic effects in FSGS may extend beyond its role in depleting B-cells. Rituximab may exert its protective effects by modulating SMPDL3b expression, thereby enhancing podocyte resistance to the damage seen in FSGS. Together, these studies suggest that targeting pathways involving SMPDL3b could be a promising strategy for treating FSGS, potentially through therapies like rituximab [50].

## 5. The Role of Sphingolipids in Diabetic Kidney Disease

Diabetic kidney disease (DKD) is the prevailing complication of diabetes and the leading cause of kidney failure worldwide [76]. It is considered that DKD results from the progression of systemic and local injuries clinically manifested by impaired renal function, with or without elevated albumin concentration in the urine. It has also been closely associated with podocyte dysfunction, which plays a critical role in the advancement of end-stage kidney disease (ESKD) [77]. Compelling evidence has identified sphingolipids as potential drivers of kidney pathology involved in numerous features of DKD. Until recently, most research was conducted on animal models, suggesting that hexosylceramides and lactosylceramides are produced and accumulate in the kidney, causing organ damage. However, recent human studies indicate that circulating sphingolipids and glycosphingolipids may also mirror or predict kidney damage [52,78]. Moreover, the loss of podocytes in patients with DKD and changes in the SL composition of podocytes have been shown to contribute to the development and progression of DKD [79]. However, more studies are still needed before SL concentrations can be used for prediction of disease occurrence and progression.

A growing number of studies have linked the role of ceramides across the entire spectrum of metabolic syndrome [80,81,82,83]. The buildup of ceramides directly causes insulin resistance by disrupting insulin signaling pathways and decreasing glucose transport. Additionally, abnormalities in ceramides and other sphingolipids lead to mitochondrial dysfunction and oxidative stress [84]. Besides ceramides, other sphingolipids, such as sphingomyelin and glucosylceramide, inhibit insulin action [85]. Some studies have demonstrated that diabetic patients exhibit increased plasma levels of sphingosine, ceramide, and glycosphingolipids, suggesting dysregulation of SL metabolism [47,53,86]. Elevated serum sphingomyelin levels have been correlated with an increased risk of ESKD in patients with type 1 diabetes (T1D), while lipidomic analysis did not show significance for ESKD but certain SM species were correlated with all-cause mortality [87,88]. 

Sphingosine-1-phosphate (S1P) maintains normal podocyte function by promoting cell growth and survival [55]. Insulin signaling influences S1P levels, as demonstrated by insulin injections preventing S1P increase in streptozotocin (STZ)-treated mice glomeruli [89]. Interestingly, it was shown that diabetic patients have decreased serum levels of S1P and related sphingolipids compared to healthy individuals, with S1P levels correlating with T2D progression and cardiovascular complications [90].

Numerous studies on animal models have demonstrated the role of S1P metabolism disorders in the progression of kidney disease. In the kidney, S1P has different mechanisms of action depending on the source and site of action. Extracellular S1P is a ligand for five receptors, S1PR1-5, which activate various intracellular cascades in processes such as migration and apoptosis. Expression of all S1PR types has been found in kidney studies; however, for S1PR5 in mouse kidneys there are divergent data [91]. S1PR4 is present in mice but has not been found in human podocytes [21]. Changes in S1PR2 and S1PR1 expression in diabetic rats contribute to DKD progression, with S1PR2 inhibition and S1PR1 activation showing protective effects against renal injury in mouse models [92]. S1PR1 activation also reduces ischemia-reperfusion injury in mouse kidneys [93]. Intracellular S1P acts independently of S1P receptors and serves as a cellular mediator in several different functions that can have similar or opposite effects on cellular processes compared to extracellular S1P. Disorders in S1P metabolism lead to dysregulation of inflammation and fibrosis processes in the perivascular cells of the kidney [94]. Due to its effects on the endothelium, S1P signaling can have a positive impact not only on the kidney but also on coronary function. Moreover, the study by Xu et al. investigated the effects of the S1P receptor agonist FTY720 (fingolimod) on coronary flow reserve (CFR) in diabetic rats. DKD is often accompanied by cardiovascular complications, including impaired coronary microcirculation, which can be reflected in a reduced CFR. The study finds that treatment with FTY720 restores CFR in diabetic rats, suggesting a protective effect on coronary circulation. The mechanism behind this involves the modulation of sphingolipid metabolism, particularly the S1P signaling pathway, which is known for its roles in cell survival, inflammation, and vascular function. By activating S1P receptors, FTY720 helps to counteract the endothelial dysfunction and microvascular damage typically observed in diabetes. This not only improves coronary blood flow but also highlights the potential therapeutic benefits of targeting sphingolipid pathways in managing diabetic complications, including both cardiovascular and kidney-related outcomes [95].

Although the function of C1P in renal disorders is less known than that of ceramide and S1P, based on the findings, it is evident that C1P deficiency is associated with the development of DKD and that targeting C1P could be a unique approach to treating patients with DKD [21,96]. 

In studies focusing on the role of sphingolipid signaling in glomerular diseases, particularly on DKD and FSGS, using ob/ob and db/db mice as models. These mice are commonly used to study obesity and T2D due to their genetic mutations leading to leptin deficiency (ob/ob) or leptin receptor dysfunction (db/db), which result in insulin resistance, obesity, and eventually, DKD. Increased levels of glucosylceramide were observed in the kidneys of both diabetic ob/ob mice and STZ-induced diabetic rats, while lactosylceramide levels rose in diabetic db/db mice [97,98]. This highlights that altered sphingolipid metabolism, especially increased levels of ceramide, contributes significantly to the development of glomerular damage seen in DKD. This damage includes podocyte injury, inflammation, and fibrosis, which are hallmarks of both DKD and FSGS [54]. Studies of plasma from T1D patients reveal that reduced long and very-long lactosylceramides are significantly associated with a higher risk of developing macroalbuminuria [78]. Gangliosides, which are glycosphingolipids with sialic acid side chains, are crucial in maintaining glomerular function and modulating signal transduction [99]. Increased monosialodihexosyl (GM3) gangliosides are found in the kidney cortices of diabetic rats and DKD patients, suggesting a link to DKD pathogenesis [21]. Serum levels of sialic acid-gangliosides positively correlate with hemoglobin A1c, blood glucose, serum creatinine, and urea levels, and microalbuminuria in individuals with DKD [100]. The aforementioned studies suggest that glycosphingolipids are crucial for maintaining proper podocyte function and preventing the progression of DKD.

Thus, changes in sphingolipids through various potential mechanisms, or a combination of these mechanisms, may play a role in the complications of diabetes, particularly in the onset of DKD.

## 6. Sphingolipids in Kidney Transplantation

Despite the advances in understanding the sphingolipid functions and alterations in cardiovascular, metabolic, and some kidney diseases, abnormalities of the SL metabolism and function after kidney transplantation remain largely unexplored. Most attention to the role of sphingolipids in kidney transplantation was given after the discovery of several potent immunosuppressants acting on the SL metabolism. The first was myriocin, an inhibitor of serine palmitoyltransferase, which, however, had significant toxicity but has paved the way for the evolution of drugs affecting SL pathways and their effects on the immune system. Through further development, fingolimod (also known as FTY720), an S1PR modulator, was discovered and developed for use as an immunosuppressant [101]. Immunosuppressive effects of FTY720 are due to the role of S1P in lymphocyte egress, and, in the case of myriocin, inhibition of T-cell proliferation. Due to inconsistent results, fingolimod was not adopted for use in kidney transplantation but is, however, approved for use in the treatment of multiple sclerosis [101,102]. Since T-cells are a part of the pathogenesis of ischemia-reperfusion injury, T-cell depletion by S1P agonism was postulated to be a possible target for its treatment and prevention. Research in mouse and rat models has shown a protective role of fingolimod in ischemia-reperfusion injury, and the studies have demonstrated increased kidney antioxidant capacity, and reduced signs of inflammation and lymphocyte infiltration. These findings have postulated the possible use of S1P agonists in protecting the kidney from acute kidney injury [103,104]. Considering that the transplantation event, particularly cadaveric, is associated with ischemia-reperfusion injury, these findings open an important avenue for further research.

An important area of transplantation research involves the studies of recurrent glomerular disease after transplantation, the most frequent of which, and with the worst outcomes, is the recurrence of focal segmental glomerular sclerosis (FSGS) [105]. FSGS often leads to a recurrence of proteinuria in about one-third of patients with FSGS after kidney transplantation. Sphingomyelinase-like phosphodiesterase 3b (SMPDL-3b) is an enzyme controlling the activity of sphingomyelinase, and its increased expression in the native kidney has been associated with protective effects against radiation injury, but also with disorders of insulin signaling in podocytes [75,106,107]. Fornoni et al. have demonstrated decreased SMPDL-3b in patients with FSGS recurrence post-transplantation, and also that treatment with rituximab partially prevents SMPDL-3b downregulation and podocyte apoptosis, and is also associated with a lower chance of proteinuria after transplantation [50]. 

Published research shows promising results regarding the role of sphingolipids in kidney transplantation, and future studies may help in the elucidation of disease mechanisms, possible biomarkers, and the development of novel treatment options for various conditions. Additionally, since cardiovascular diseases and metabolic disorders represent a major morbidity and mortality cause in kidney transplant recipients, discoveries regarding sphingolipids in these areas will likely drive changes in understanding and treating these disorders in kidney transplant recipients.

## 7. Conclusions

This review highlights the critical roles that SLs play in kidney function and disease, with a particular focus on CKD and DKD. This review underscores the importance of ceramides, S1P, and other SL metabolites in regulating key cellular processes such as apoptosis, oxidative stress, and inflammation, all of which are central to kidney health and disease progression. Dysregulation of SL metabolism is associated with renal injury and disease progression, as seen in the accumulation of pro-apoptotic ceramides during renal injury and the protective role of S1P in maintaining renal function. Clinically, these findings suggest that targeting SL metabolism could offer novel therapeutic strategies for CKD and DKD. For example, modulating ceramide levels or enhancing S1P signaling may help to prevent or slow the progression of kidney disease. Furthermore, SLs, particularly HDL-bound S1P, may serve as valuable biomarkers for early diagnosis, disease monitoring, and treatment response in CKD and DKD. The understanding of SLs in renal pathology is still evolving, but the evidence presented in this review emphasizes their potential as both therapeutic targets and biomarkers, offering promising avenues for improving patient outcomes in kidney disease.

## Figures and Tables

**Table 1:** Summary of selected sphingolipid alterations in chronic kidney disease etiologies.

Injury Type/Disease	Model	Pathway/Phenotype	Sphingolipid	Reference
Nephrotoxic drugs/irradiation/bacterial toxin	In vitro and in vivo studies	Mitochondrial membrane permeability/ROS generation → apoptosis/cell survival	Ceramide ↑ and S1P ↓	Ueda N [24]
Renin–angiotensin–aldosterone system	Mice and in vitro	Second messenger/Rho–kinase pathway; vascular dysfunction (S1P); aldosterone synthesis	Ceramide; S1P	Berry et al. [48]; Meissner et al. [49]
Podocyte dysfunction				
Focal segmental glomerulosclerosis	Human	Actin cytoskeleton remodeling, apoptosis	SMPDL3b overexpression; GM3 ↓, S1P (?)	Fornoni et al. [50]; Drexler et al. [51]
Diabetic kidney disease	In vitro, mice, in vivo	Podocyte insulin signaling dysregulation; podocyte dysfunction; promotion of fibrosis	iS1P ↑, iGM3 ↓, iC1P ↓, ceramide ↑, SMPDL3b activity ↑	Hammad et al. [52]; Gorska et al. [53]; Malella et al. [21]; Mitrofanova et al. [54]
S1P lyase deficiency	Human, mice, rats	Sphingolipid accumulation, foot process effacement	S1P ↑, ceramide ↑	Schumann et al. [55]; Imeri et al. [56]
Farber disease	Human	Foot process effacement	Ceramide ↑	Li et al. [57]
Niemann-Pick disease	Human	Lipid accumulation, glomerular sclerosis	Sphingomyelin ↑	Grafft et al. [58]
Fabry disease	Human	Increased autophagy, mTOR dysregulation (?)	Gb3 ↑	Liebau et al. [59]
Gaucher disease	Human	Podocyte dysfunction, Gaucher cell formation	Glucosyceramide	Santoro et al. [60]

Note—(?) indicates suspected or most likely alterations which are not yet fully confirmed; S1P—Sphingosine-1-phosphate; SMPDL3b—sphingomyelinase-like phosphodiesterase 3b; GM3—a ganglioside type (monosialodihexosylganglioside); i—intracellular; C1P—ceramide-1-phosphate; mTOR—mammalian target of rapamycin; Gb3—globotriaosylceramide; ↑ increased concentration/accumulation; ↓ decreased concentration .
